# 

**Published:** 1933

**Authors:** 


### The Medical Library of the University of Bristol,

WITH WHICH IS INCORPORATED

**The Library of the Bristol Medico-Chirurgical Society.**

*The following donations have been received since the publication of the list in February,* 1933.

June, 1933.

| | |
|---|---|
| W. S. Bainbridge, Esq. (1) .. .. .. | 1 volume. |
| Dr. W. L. Cossham (2) .. .. .. .. | 2 volumes. |
| Director of the Commission on Medical Education (3) .. .. .. .. .. | 1 volume. |
| E. Wilfred Fish, Esq. (4) .. .. .. | 2 volumes. |
| General Medical Council (5) .. .. .. | 3 ,, |
| Glasgow Royal Maternity and Women's Hospital (6) .. .. .. .. .. | 1 volume. |
| Professor E. W. Hey Groves (7) .. .. | 7 volumes. |
| London School of Hygiene and Tropical Medicine (8) .. .. .. .. .. | 1 volume. |
| Michell Clarke (9) .. .. .. .. .. | 1 ,, |
| Middlesex Hospital (10) .. .. .. .. | 1 ,, |
| Dr. George Parker (11) .. .. .. .. | 2 volumes. |
| John Wright & Sons Ltd. (12) .. .. .. | 15 ,, |

Unbound periodicals have also been received from Professor E. W. Hey Groves.

### THE ONE HUNDRED AND FORTY-FOURTH LIST OF BOOKS.

The figures in round brackets refer to the figures after the names of the donors. The books to which no such figures are attached have either been bought from the Library Fund or received through the *Journal.*

| | | | |
|---|---|---|---|
| *Académie Royale de Chirurgie.* | *Deuxième Cententenaire* .. .. | (7) | 1931 |
| **Bardswell, N. D.** .. | *Advice to Consumptives* .. .. .. .. | (12) | 1910 |
| **Bowlby, A.** .. .. | *Wounds in War* .. .. .. .. .. .. | (12) | 1916 |
| **Boyd, R. H.** .. .. | *Diet and Care of the Surgical Case* .. .. | | 1930 |
| **Braithwaite, J. V. C.** | *Infant Feeding in General Practice* .. .. | | 1930 |
| **Brooke, R.** .. .. | *Shorter Orthopædic Surgery* .. .. .. .. .. | | 1932 |
| **Clark, A. J.** .. .. | *Mode of Action of Drugs on Cells* .. .. | | 1933 |
| **Clock, R. O.** .. .. | *Our Baby* .. .. .. .. .. .. .. .. | (12) | 1912 |
| **Crawford, A. M.** .. | *Materia Medica for Nurses* .. .. .. .. .. | | 1933 |
| **DeLee, J. B. and Greenhill, J. P.** | *Obstetrics and Gynecology* .. .. | | 1931 |
| **Dieudonné, A.** .. .. | *Bacterial Food Poisoning* .. .. .. | (12) | 1909 |
| **Douglas, A. C.** .. .. | *Physical Mechanism of the Human Mind* | (2) | 1932 |
| **Fenwick, P. C.** .. | *Diseases of the Urethra* .. .. .. .. | (12) | 1906 |
| **Fish, E. W.** .. .. | *Experimental Investigation of Enamel Dentine and the Dental Pulp* .. .. | (4) | 1933 |
| **Fish, E. W.** .. .. | *Principles of Full Denture Prosthesis* .. | (4) | 1933 |
| **Flesch-Thebesius, M.** | *Chirurgische Tuberkulose* .. .. .. | (7) | 1933 |
| **Francis, A.** .. .. | *Treatment of Asthma* .. .. .. .. .. .. | | 1932 |
| **Gabriel, W. B.** .. .. | *Principles and Practice of Rectal Surgery* .. | | 1932 |
| **Gray, H. M. W.** .. | *Early Treatment of War Wounds* .. .. | (12) | 1919 |
| **Gullan, M. A.** .. .. | *Theory and Practice of Nursing* .. | 3rd Ed. | 1930 |
| **Haldane, J. S.** .. .. | *Philosophical Basis of Biology* .. .. | (11) | 1931 |
| **Hale-White, W.** .. | *Materia Medica* .. .. .. .. .. | 21st Ed. | 1932 |
| **Haour, J.** .. .. .. | *Données Actuelles sur la Régénération Osseuse Aseptique Chez l'Adulte* .. .. .. | (7) | 1919 |
| **Hewer, G. L.** .. .. | *Recent Advances in Anæsthesia and Analgesia* | | 1932 |
| **Holland, E., and others** | *A Doctor to a Mother* .. .. .. .. .. .. | | 1933 |
| **Körte, Werner** .. .. | *Die Erkrankungen der Gallenwege* .. | (12) | 1928 |
| **Lagarde, C. L. A.** .. | *Gunshot Injuries* .. .. .. .. .. .. | (12) | 1914 |
| **Lambert, G.** .. .. | *Cardiovascular Pain as a Biochemical Problem* | | 1933 |
| **Lang** .. .. .. .. | *German–English Dictionary.* Edited by M. K. Meyers .. .. .. .. .. | 4th Ed. | 1932 |
| **Laquer, F.** .. .. | *Hormone und innere Sekretion* .. .. | (12) | 1928 |
| **Levi, D.** .. .. .. | *Injection Treatment of Varicose Veins* .. | | 1932 |
| **Lewis, T.** .. .. .. | *Diseases of the Heart* .. .. .. .. .. .. | | 1933 |
| **London School of Hygiene and Tropical Medicine.** | *Handlist of Periodicals in the Library* .. .. .. | (8) | 1933 |
| **Love, R. J. M.** .. | *A Shorter Surgery* .. .. .. .. | 3rd Ed. | 1932 |
| **Lund, J. K.** .. .. | *Diabetics* .. .. .. .. .. .. .. .. .. .. | | 1929 |
| **MacCallum, W. G.** .. | *Textbook of Pathology* .. .. .. | 5th Ed. | 1932 |
| **Martin, C. R. A.** .. | *Practical Food Inspection* Vol. I... .. | | 1932 |
| **McCleary, G. F.** .. | *Early History of the Infant Welfare Movement* | | 1933 |

**Miles, A., and Wilkie, D. P. D.** *Operative Surgery* .. .. .. .. .. 1933
**Moore, C. N.** .. .. *Nutrition of Mother and Child* .. .. (12) 1923
*Nouveau Traité de Médecine.* Vol. X., Part 3 .. .. .. .. .. (9) 1933
**Öhnell, H.** .. .. .. *Die Magengeschwärs Krankheit* .. .. (12) 1929
**Papayoannou, T. L.** *Livre d'or à l'Occasion du Jubilé de Vingt-cinq ans d'Activité Chirurgicale du Docteur T. L. Papayoannou* .. .. .. .. .. (7) 1932
**Pearson, W. J., and Watkins, A. G.** *The Infant* .. .. .. .. .. 1932
**Quervain, F. De.** .. *Kurzgefasste lehre von den Knochenbrächen* (7) 1913
**Robertson, J. D.** .. *Gastric Acidity* .. .. .. .. .. .. .. (10) 1931
**Robinson, I.** .. .. *Baldness and Greyness* .. ..2nd Ed. (12)
**Roxburgh, A. C.** .. *Common Skin Diseases* .. .. .. .. .. .. 1932
**Russell, W. K.** .. *Colonic Irrigation* .. .. .. .. .. .. (2) 1932
**Simmonds, R. M.** .. *Handbook of Diets* .. .. .. .. .. .. .. 1931
**Smart, M.** .. .. .. *Principles of Treatment of Muscles and Joints by Graduated Muscular Contractions* 1933
**Smith, G. E., and others.** *Early Man* .. .. .. .. .. .. .. (11) 1931
**Swanberg, Harold** .. *Radiologic Maxims* .. .. .. .. .. .. .. 1932
**Tidy, N. M.** .. .. *Massage and Remedial Exercises* .. .. .. 1932
**Tindal, D.** .. .. .. *Gleanings from General Practice* .. .. .. 1929
**Tomson, W. B.**.. .. *Prevention of Tuberculosis* .. .. .. .. .. 1929
**Von Neusser, E.** .. *Clinical Treatises on Respiration and Circulation* .. .. .. .. .. .. .. (12) 1907
**Wittie, R.** .. .. .. *Scarbrough Spaw* . . . .. .. .. .. .. 1660

### TRANSACTIONS, REPORTS, JOURNALS, ETC.

Dentists' Register, 1933 .. .. .. .. .. .. .. .. .. .. .. .. .. (5) 1933
Glasgow Royal Maternity and Women's Hospital. Medical Report for 1931 .. .. .. .. .. .. .. .. .. .. .. .. .. .. .. (6) 1932
Medical and Dental Students' Register, 1932 .. .. .. .. .. (5) 1932
Medical Education, Final Report .. .. .. .. .. .. .. .. .. .. (3) 1932
Medical Register, 1933 .. .. .. .. .. .. .. .. .. .. .. .. .. (5) 1933
Ophthalmic Yearbook, XXI. .. .. .. .. .. .. .. .. .. .. .. (12) 1925
Quarterly Cumulative Index Medicus. Vol. 12 .. .. .. .. .. .. 1932
Report on Public Health and Medical Subjects, No. 11 .. .. (12) 1922
Report on Sixth International Congress of Military Medicine and Pharmacy .. .. .. .. .. .. .. .. .. .. .. .. .. .. .. .. (1) 1933
Société Internationale de Chirurgie. IX. Congres. 2 Vols. .. .. 1932
Surgical Clinics of North America. Vol. 12, No. 6 .. .. .. .. .. 1932
Surgical Clinics of North America. Vol. 13, No. 1 .. .. .. .. .. 1933
Transactions of the Ophthalmological Society of the United Kingdom. Vol. III. .. .. .. .. .. .. .. .. .. .. .. .. .. .. .. .. 1933

### Publications Received

---

From Baillière, Tindall & Cox. :

*The Medico-Legal and Criminological Review.* Vol. I. Price 3s. 2d.

*The Common Causes of Chronic Indigestion.* By Thomas C. Hunt, B.A., D.M., M.R.C.P. Price 12s. 6d.

From John Bale, Sons and Danielsson Ltd. :

*Cure of Hæmorrhoids, Varicose Veins and Ulceration.* By Stuart McAusland, B.A., M.D., Ch.B., M.R.C.S., L.R.C.P. Price 3s. 6d.

From Bureau Tot Bevordering Van Het Kinine-Gebruik.

*Chinin-Formeln.* 3rd Ed. 1933.

*Chinin in der Allgemeinpraxis.* Edited by Fritz Johannessohn. 1932.

From The Commonwealth Fund :

*Standard Classified Nomenclature of Disease.* Edited by H. B. Logie, M.D., C.M.

From William Heinemann Ltd. :

*Natural Childbirth.* By Grantly Dick Read, M.A., M.D. Price 7s. 6d.

*Psychology of Sex.* By Havelock Ellis. Price 12s. 6d.

*Vitamins and other Dietary Essentials.* By W. R. Aykroyd, M.D. Price 7s. 6d.

*A Surgeon's Pocket-Book.* By H. S. Souttar, D.M., M.Ch. Price 7s. 6d.

From H. K. Lewis & Co. Ltd. :

*Cardiovascular Pain as a Biochemical Problem.* By Gordon Lambert, B.A., M.D., B.C. Price 6s.

*Simple Instructions for Diabetic Patients.* By Dorothy C. Hare. Price 1s.

*Internal Derangements of the Knee Joint.* By A. G. T. Fisher, M.C., M.B., Ch.B. Price 15s.

*Clinical Ophthalmology.* By J. M. Bickerton, M.A., B.Ch., and L. H. Savin, M.D. Price 7s. 6d.

*Treatment of Functional Nerve Cases by the Method of Neuro-Induction.* By Leonard Inkster.

*Sanitary Inspector's Handbook.* By H. H. Clay. Price 15s.

*Adjustment of Muscular Exercise.* By J. K. McConnel, M.C. Price 4s. 6d.

*Studies on the Physiology of the Eye.* By J. G. Byrne. Price 40s.

*Practical Food Inspection.* Vol. II. By C. R. A. Martin. Price 10s. 6d.

From OXFORD UNIVERSITY PRESS:

*Principles of Treatment of Muscles and Joints by Graduated Muscular Contractions.* By MORTON SMART, C.V.O., D.S.O., M.D., Ch.B. Price 15s.

*Inherited Abnormalities of the Skin and its Appendages.* By E. A. COCKAYNE, D.M., F.R.C.P. Price 32s.

*Operative Surgery.* By A. MILES, M.D., LL.D., and D. P. D. WILKIE, M.D. Price 21s.

*Surgical Operations.* By E. W. HEY GROVES, M.D., B.Sc., M.S. Price 18s.

*Neurological Effects of Syphilis.* By BRYAN BUCKLEY SHARP, M.D. Price 7s. 6d.

From THE ROYAL BERKSHIRE HOSPITAL:

*Royal Berkshire Hospital Reports for* 1932 *and* 1933. Edited by H. S. LE MARQUAND. Price 10s. 6d. each.

From STUDENT CHRISTIAN MOVEMENT PRESS:

*The Christian Faith To-day.* Price 2s. 6d.

From JOHN WRIGHT & SONS LTD.:

*The Liver Diet Cookery Book.* By DOROTHY SEWART. Price 1s. 6d.

*An Outline of Medicine for Nurses.* By JAMES FANNING, M.D., D.P.H. Price 2s. 6d.

*The Injured Workman.* By G. F. WALKER, M.D., M.R.C.P. Price 6s.

*Synopsis of Surgery.* By E. W. HEY GROVES, M.S., M.D., B.Sc.

*British Journal of Surgery.* Vol. No. 80. Price 12s. 6d.

*The Medical Annual,* 1933. Edited by the late C. F. COOMBS and A. R. SHORT. Price 20s.

*Demonstrations of Physical Signs in Clinical Surgery.* 9th Ed. By HAMILTON BAILEY. Price 21s.

### Local Medical Notes

EXAMINATION RESULTS.

**University of Bristol.**—Students of the University have recently passed the following examinations :—

M.B., Ch.B.—*Second Examination.* (*Part II.*) *Pass :* Ursula G. Hewitt, L. A. Schnipelsky, E. Want, P. Zimmering.

M.B., Ch.B.—*Final Examination.* (*Part I. Including Forensic Medicine and Toxicology.*) *Pass :* R. D. Bodman, J. R. G. Damrel, F. J. W. Lewis, S. P N. Williams. *Part I. only :* M. W. Maged.

M.B., Ch.B. — *Final Examination.* (*Part II.*) *Pass* (*Second Class Honours*) *:* A. J. Board (with Distinction in Public Health). *Pass :* J. S. Adamson, C. J. N. Davis (with Distinction in Obstetrics), G. M. Evans, Winifred M. Hill, R. L. Marks, H. E. Pearse. *Group I. only :* Aldyth D. Jones, F. E. Powell.

B.D.S.—*Second Examination. Pass : In Anatomy and Physiology only :* A. N. Brimelow, P. R. Quartley.

B.D.S.—*Third Examination. Pass :* J. W. E. Snawdon (with Distinction in Dental Mechanics, Dental Metallurgy and Dental Materia Medica).

L.D.S.—*Preliminary Science Examination. Pass : In Chemistry* (*Completing Examination*) *:* H. D. Williams. *In Chemistry and Physics only :* J. B. Inverdale and H. W. Williams.

L.D.S.—*First Professional Examination. Pass : In Dental Anatomy* (*Completing Examination*) *:* R. E. Buchan. *In Anatomy and Physiology* (*Completing Examination*) *:* J. G. Coates, R. A. Hilton, T. John, C. L. Read, A. J. Staple. *In Dental Anatomy only :* M. J. Banbury.

L.D.S.—*Second Professional Examination. Pass* (*Dental Materia Medica only*) *:* B. E. Ffrench, Frances M. Orton, L. E. Scull.

L.D.S.—*Final Professional Examination. Pass :* I. R. R. Eskell, J. Hewitt, J. H. G. Smith.

**Conjoint Board.**—M.R.C.S., L.R.C.P.—*Pass : In Physics :* A. C. F. Alexander. *In Anatomy and Physiology :* J. A. Cochrane. *In Pharmacology and Materia Medica :* C. F. Rivington and L. E. Sealy. *In Medicine :* J. L. S. James.* *In Medicine and Surgery :* Edith K. Harris.* *In Pathology :* Winifred M. Hill, F. L. Wollaston and C. H. G. Price. *In Surgery :* G. L. Foss and S. B. Adams.

L.D.S., R.C.S.—*First Professional Examination. Pass : Part II. (Dental Metallurgy) :* D. J. Dymond, J. G. Jones. *Part III. (a) (Anatomy and Physiology) :* J. T. S. Hutchins. *Part III. (b) (Dental Anatomy and Physiology) :* R. Edwards, L. Jonathan, W. B. Lewis.

* Qualified.

